# Research on Forging Process of C83600 Tin Bronze Valve Body Based on Rheological Behavior and Hot Processing Diagram

**DOI:** 10.3390/ma18122872

**Published:** 2025-06-17

**Authors:** Jian Yang, Yangbiao Zeng, Yuhang Chen, Lirong Huang, Wen Liu, Chaoyang Wang, Xiao Qin

**Affiliations:** 1 Guangdong Chengtai Automation Technology Co., Ltd., Huizhou 516000, China; 2 School of Mechanical and Electrical Engineering, Jiangxi University of Science and Technology, Ganzhou 341000, China; 3 School of Information and Intelligent Engineering, Zhejiang Wanli College, Ningbo 315100, China; 4 Ningbo Huacheng Valve Co., Ltd., Ningbo 315100, China; 5 Mengyin County Peng Cheng Wan Li Vehicle Co., Ltd., Linyi 276200, China

**Keywords:** hot compression forming, Arrhenius constitutive equation for strain compensation, rheological behavior, thermal processing diagrams, tin bronze body

## Abstract

To achieve high-performance forgings of the C83600 tin bronze valve body with a uniform structure that is free from forging defects, rheological data were collected via hot compression experiments. Subsequently, an Arrhenius constitutive model incorporating strain compensation was established. The correlation coefficient, root mean square error, and mean relative error between the predicted values of the model and the experimental results were 0.99326, 5.1898, and 4.022%, respectively, which validated the model’s capability to accurately describe the rheological behavior of C83600. Using this model, the rheological data were incorporated into the Deform material library to enhance its database. A thermal processing map for C83600 under various deformation conditions was then developed. This map indicates that the material demonstrates excellent thermal working stability when the deformation temperature ranges from 850 to 900 K and the strain rate varies between 0.0067 and 0.0483 s^−1^. Furthermore, numerical simulations were conducted to analyze the forging process, focusing on regions of stress concentration where the average strain rate aligns with the optimal parameters derived from the thermal processing map. This alignment not only verifies the reliability of the hot working map but also confirms the feasibility of the forging process through trial production.

## 1. Introduction

The valve body is the core component of a valve, comprising over half of its total mass. In oil and gas exploitation and transmission systems, the valve body must meet elevated requirements for corrosion and pressure resistance due to varying pressures, temperatures, and potential corrosion in different application environments [1]. Copper alloys, known for their superior corrosion resistance compared to cast iron, are commonly used in valves. Tin bronze, in particular, exhibits high chemical stability in diverse environments including the atmosphere, water vapor, freshwater, seawater, and alkaline solutions [2].

Tin bronze demonstrates exceptional fluidity and a low shrinkage rate, making it highly suitable for the casting of complex components. Dong et al. [3] conducted a comprehensive analysis of the casting characteristics of tin bronze. With a focus on the structural design of the two-way plug valve shell, they investigated potential casting defects, including shrinkage porosity and shrinkage cracking, that are likely to occur during production. Furthermore, an in-depth evaluation of the casting processes for the two types of plug valve shells was performed. By implementing the proposed casting process, multiple batches of high-quality plug valve shell castings were successfully manufactured. This approach effectively maintained a low pump leakage rate, thereby fulfilling the requirements for mass production. Wei et al. [4] conducted a comprehensive analysis of the structural design of the four-valve box, identifying potential issues and elaborating on the corresponding casting process characteristics. Through the implementation of appropriate measures, the leakage of the four-valve box was successfully mitigated to a relatively low level, thereby fulfilling the requirements for mass production. Xiao Zhu et al. [5] developed a top-pouring gating system by considering the thermophysical properties of tin bronze (C83600) and the geometric features of thin-walled valve body castings. They investigated the casting process parameters, optimized the gating system through modifications to the heat dissipation method and the application of orthogonal experiments, and evaluated the quality of castings under different casting processes. The findings indicate that refining the design of the gating system, along with adjusting the appropriate pouring temperature, pouring rate, and preheating temperature of the sand mold, can significantly enhance the quality of the castings and promote their successful one-time formation.

However, this alloy exhibits a significant tendency for suction, and molten copper often “rises” in the last places to solidify, such as in gates and risers [6]. Under typical casting conditions, castings exhibit compositional non-uniformity due to segregation and diminished mechanical properties and air tightness due to numerous small internal pores [7]. However, its minimal volume shrinkage rate during solidification allows for the casting of parts with uneven wall thicknesses and complex shapes using only small risers [8]. Post-casting machining of the tin bronze valve body often reveals tiny pockmarks under the microscope, resembling “fly feet”, and leaks can occur under high pressure [9], failing to meet long-term performance requirements in high-pressure and corrosive conditions.

Forging significantly enhances the mechanical properties of tin bronze castings, refines the grain structure, and simultaneously improves the material’s toughness, corrosion resistance, and wear resistance. During forging, the physical and mechanical properties of tin bronze are substantially affected by temperature variations. Establishing an appropriate forging temperature is crucial for ensuring optimal material performance. The forging temperature can be precisely determined through the analysis of the alloy’s thermal deformation behavior, the construction of thermal working diagrams, and the implementation of numerical simulations of the forging process. Sellars et al. [10] were pioneers in proposing a constitutive equation that quantitatively describes the relationship among flow stress (σ), deformation temperature (T), and strain rate (ε) during thermal deformation. Current research on constitutive equations describing the thermal deformation behavior of metals encompasses the Arrhenius model [11], Johnson–Cook model [12], and Avrami model [13]. Sun Honglei et al. [14] developed a strain-compensated Arrhenius constitutive model for the HPb59-1 copper alloy and evaluated its prediction accuracy using the correlation coefficient and average absolute error. Finite element simulation technology, as demonstrated by He Lianfang [15] using Deform-3D (V11.0) software, has proven to be effective in improving the quality of forged parts and reducing research and development costs. Similarly, Gaogui [16] addressed issues such as incomplete filling at the handle end of a valve body and the tendency for marks to fall off, thereby reducing the maximum forming force by 17.5% and increasing material utilization by 7.2%.

In summary, to enhance the casting performance and satisfy long-term performance requirements of tin bronze under high-pressure and corrosive conditions, forging can be utilized as an effective method to improve mechanical properties and ensure the air-tightness of castings. Numerical simulations are conducted to determine optimal forging parameters, validate the constitutive equation, and construct the hot working diagram. Research on the forging process of thin-walled tin bronze valve bodies includes establishing a constitutive equation and developing a hot working diagram for the C83600 tin bronze alloy based on data obtained from hot compression experiments, thereby providing guidance for its forging process.

## 2. Experiment and Method

### 2.1. Hot Compression Experiment

**Figure 1:**
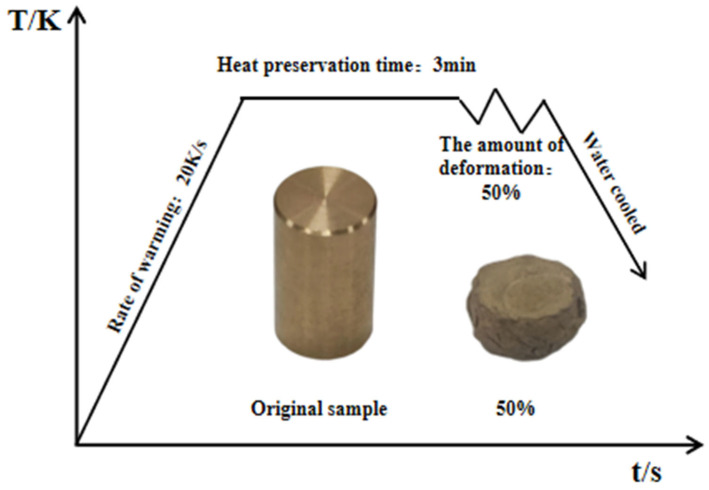
Flow chart of hot compression tests.

The company Ningbo Huacheng Valve Co., Ltd., (Ningbo, China). supplied the experimental materials, specifically C83600 tin bronze, along with the relevant process parameters for establishing the deformation conditions in the hot compression experiment. Additionally, they provided the experimental equipment required for subsequent valve forging. Further details are provided in Table 1. Isothermal hot compression tests were conducted using a Gleeble-3500 thermal simulation testing machine (made in Fanrui Yunzhi Technology Co., Ltd., Zhengzhou, China). Graphite lubricant was evenly applied to both ends of each sample to minimize friction. Compression tests were performed at temperatures of 873 K, 973 K, and 1073 K, with corresponding deformation rates of 0.001, 0.01, 0.1, and 1 s^−1^, and a deformation amount of 50%. The samples were heated at a rate of 20 K/s. Upon reaching the target temperature, the samples were equilibrated for three minutes before compression began. After compression, the samples were immediately quenched to preserve their high-temperature microstructures. The experimental procedure is illustrated in Figure 1.

### 2.2. Finite Element Model

To assess the feasibility of the C83600 constitutive model and hot working diagram for guiding the forging process, a numerical simulation was performed. The forging analyzed was a small, thin-walled, three-way valve body. Initially, a three-dimensional model of the die and blank was created in SolidWorks (V2024) and exported in STL format, then imported into Deform software (V11.0) for preprocessing as its finite element model. The numerical simulation was conducted using the forging simulation software Deform. Leveraging the robust mesh regeneration capabilities of this software, a total mesh count of 120,000 was configured. During the subsequent simulation process, Deform can automatically re-mesh as required until the simulation is fully completed. The boundary conditions and process parameters are summarized in Table 2. In this simulation, a common graphite lubricant was employed with a friction coefficient set to 0.2. Additionally, the material mechanics model was established based on the findings of this study.

**Figure 2:**
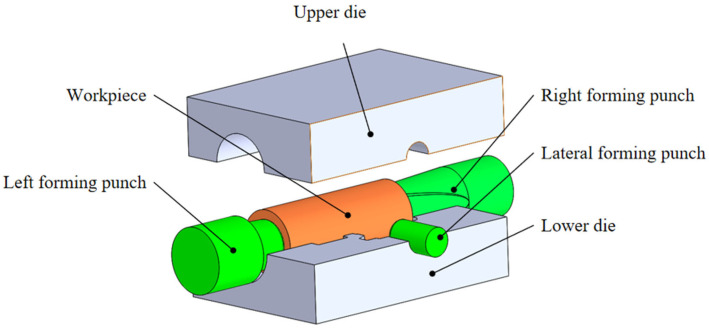
Finite element model.

**Figure 3:**
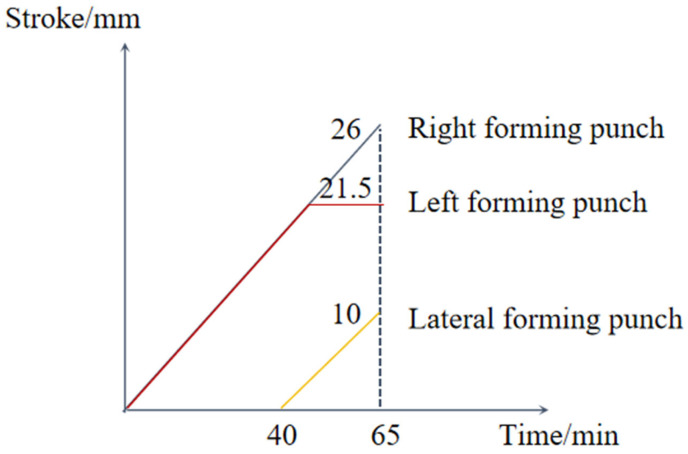
Forming punch stroke–time curve.

The finite element model is depicted in Figure 2. During the simulation, the motion of the die and forming punch followed a specific sequence: initially, both left and right forming punches moved at a designated speed to compress the blank; the left punch then held its position while the right punch continued moving. Concurrently, the lateral punch began to move at a set speed, until all components reached their designated positions to complete the forging. The stroke–time curves for each component are shown in Figure 3.

## 3. Results and Discussion

### 3.1. Rheological Curve Analysis

Friction correction and temperature rise correction were performed on the experimental data obtained from the hot compression tests. Specifically, the expansion coefficient *B_p_*, as presented in Formula (1) and proposed by Roebuck [17], was utilized to evaluate whether the stress–strain curves obtained from the experiments required friction correction.

(1) $$Bp=\frac{h_{1}r_{m}^{2}}{h_{0}r_{0}^{2}}$$

Among these parameters, *h*_0_ represents the initial height of the sample before compression; *h*_1_ denotes the height after compression; *r*_0_ indicates the radius prior to compression; and *r_m_* signifies the maximum radius before deformation. The expansion coefficient of the deformed sample under various experimental conditions is calculated using the provided formulas. It is observed that *B_p_* is less than 1.1, suggesting that friction has a negligible impact on the experiment. Consequently, the obtained stress–strain data can be used as a reliable substitute for actual results.

In addition, the Formula (2) [18] is employed to ascertain whether a temperature rise correction is necessary:

(2) $$\sigma_{t}=\frac{T}{T_{t}}\sigma$$

Among these parameters, $\sigma_{t}$ is the actual stress after temperature rise correction, *T* is the experimental temperature, *T_t_* is the set deformation temperature, and *σ* is the experimental stress. By comparing the experimental temperature measured by thermocouples embedded in the sample with the preset deformation temperature, the discrepancy is found to be negligible. Consequently, the temperature increase has a minimal effect on the deformation behavior of this copper alloy material under high-temperature conditions. In summary, the experimental data can reliably represent the actual results.

**Figure 4:**
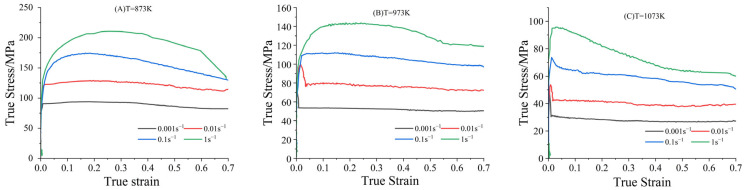
True stress–strain curve of C83600: (**A**) (873 K), (**B**) (973 K), (**C**) (1073 K).

Figure 4 illustrates the true stress–strain curve for C83600 at a consistent temperature and varying strain rates. In the micro-strain stage, work hardening occurs, and the flow stress of the alloy increases sharply. As the strain reaches a certain threshold, the curve exhibits dynamic recrystallization, and the flow stress peaks with the strain’s increment. At this juncture, the softening effect induced by dynamic recrystallization surpasses the hardening caused by deformation, leading to a decrease in stress as the strain continues to rise. The greater the degree of recrystallization, the more pronounced the softening effect and the larger the reduction in stress. Eventually, the effects of dynamic recrystallization softening and work hardening equilibrate, stabilizing the flow stress despite further increases in strain.

At a low strain rate, the flow stress curve exhibits a wavy pattern due to the low dislocation energy and slow dislocation proliferation rate characteristic of the tin bronze alloy. Following dynamic recrystallization softening, the driving force for further recrystallization diminishes, weakening the recrystallization softening effect. Consequently, this softening effect fails to balance with the new work hardening, causing the material to re-harden and the curve to ascend. As strain increases, dislocations accumulate to a critical level, allowing recrystallization to dominate and the curve to decline once more. This cyclical process of dynamic recrystallization and work hardening persists, maintaining a roughly consistent period but with gradually diminishing amplitude, resulting in the wavy alteration of the curve.

It is noteworthy that the stress–strain curves under strain rates of 0.001 and 0.01 s⁻¹, as well as deformation temperatures of 973 K and 1073 K, exhibit a significantly sharper peak compared to other deformation conditions. The stress rapidly transitions into a stable state, suggesting that these deformation conditions are more favorable for achieving a rapid equilibrium between the softening mechanism induced by dynamic recovery and the hardening mechanism caused by dislocation slip. Consequently, the stress–strain curve demonstrates a wavy variation.

### 3.2. Construction and Application of Constitutive Equation

The Arrhenius constitutive model is widely applied in high-temperature thermal deformation to describe the relationship among deformation temperature, strain rate, and flow stress [19]. The model is represented by the following equations:

(3) $$Z=\dot{\varepsilon }\exp(\frac{Q}{\mathrm{RT}})=F(\sigma )$$

where

(4) $$F(\sigma )=\left\{\begin{array}{l} \sigma^{n_{1}},\alpha \sigma <0.8\\ \exp(\beta \alpha ),\alpha \sigma >1.2\\ {\left[\sinh(\alpha \sigma )\right]}^{n},\;\mathrm{for}\;\mathrm{all}\;\alpha \sigma \end{array}\right.$$

Derived from Equations (3) and (4), Equation (5) is formulated as follows:

(5) $$\dot{\varepsilon }=\left\{\begin{array}{l} A_{1}\sigma^{n_{1}}\exp(-\frac{Q}{\mathrm{RT}}),\alpha \sigma <0.8\\ A_{2}\exp(\beta \alpha )\exp(-\frac{Q}{\mathrm{RT}}),\alpha \sigma >1.2\\ A{\left[\sinh(\alpha \sigma )\right]}^{n}\exp(-\frac{Q}{\mathrm{RT}}),\;\mathrm{for}\;\mathrm{all}\;\alpha \sigma \end{array}\right.$$

where $\dot{\varepsilon }$ denotes the strain rate (s^−1^), T represents the deformation temperature (K), σ denotes the flow stress (MPa), Q denotes the thermal deformation activation energy (KJ/mol), R denotes the gas molar constant (8.314 J/(mol∙K)), and A, *n*, *n*_1_, *β*, and *α* represent material constants, *α* = *β*/*n*_1_.

**Figure 5:**
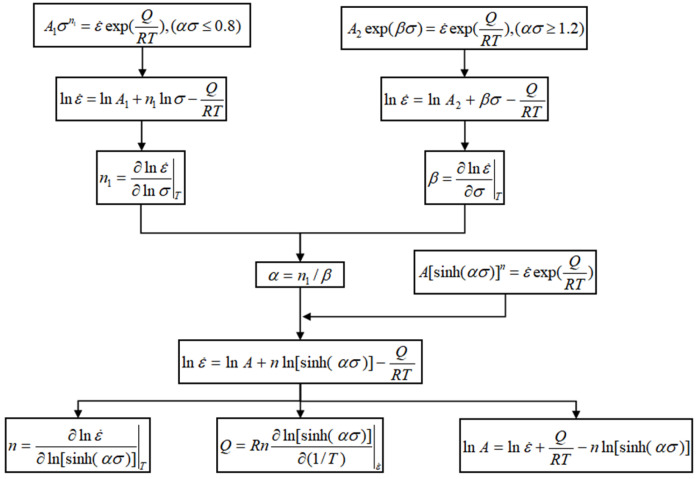
Process of calculating the material constants of the constitutive equation of Arrhenius.

**Figure 6:**
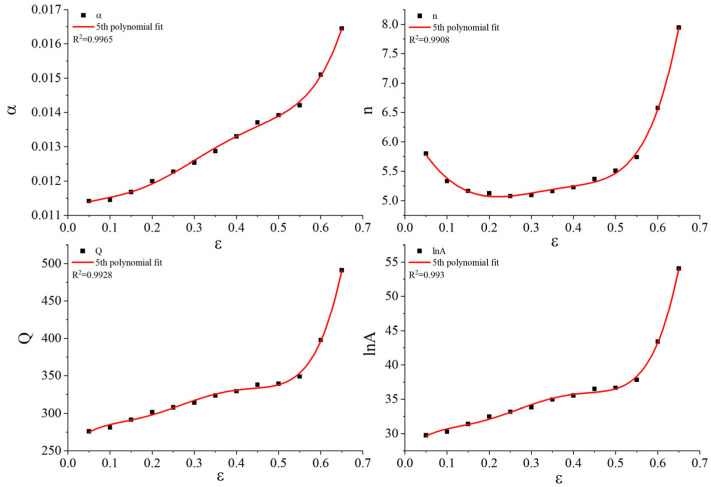
Fitting coefficients of quintic polynomial for each material constant: a, n, Q, lnA.

Based on the true stress–strain curves shown in Figure 4, material constants corresponding to 13 strain points, ranging from 0.05 to 0.65 at intervals of 0.05, were calculated as depicted in Figure 5. A quintic fitting polynomial was applied for each material constant with respect to strain, using Equation (6). As demonstrated in Figure 6, the fitting degree $R^{2}$ exceeded 0.99 for all constants, indicating high accuracy in the fitting results. The fitting coefficients of the quintic polynomial for each material constant are presented in Table 3.


(6) $$\begin{array}{l} \alpha (\varepsilon )=B_{0}+B_{1}\varepsilon +B_{2}\varepsilon^{2}+B_{3}\varepsilon^{3}+B_{4}\varepsilon^{4}+B_{5}\varepsilon^{5}\\ n(\varepsilon )=C_{0}+C_{1}\varepsilon +C_{2}\varepsilon^{2}+C_{3}\varepsilon^{3}+C_{4}\varepsilon^{4}+C_{5}\varepsilon^{5}\\ Q(\varepsilon )=D_{0}+D_{1}\varepsilon +D_{2}\varepsilon^{2}+D_{3}\varepsilon^{3}+D_{4}\varepsilon^{4}+D_{5}\varepsilon^{5}\\ \ln A(\varepsilon )=E_{0}+E_{1}\varepsilon +E_{2}\varepsilon^{2}+E_{3}\varepsilon^{3}+E_{4}\varepsilon^{4}+E_{5}\varepsilon^{5} \end{array}$$

**Figure 7:**
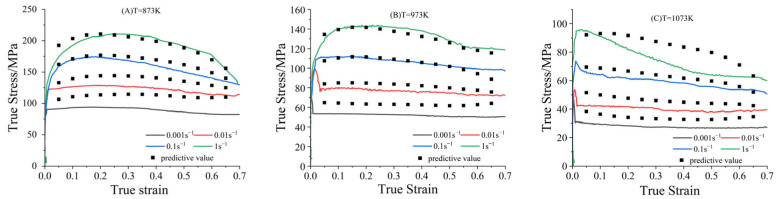
Comparison of predicted and experimental true stress–strain curves for the modified Arrhenius model: (**A**), (873 K), (**B**) (973 K), (**C**) (1073 K).

The strain-compensated Arrhenius constitutive equation, shown in Equation (7), alongside the quintic fitting polynomial jointly predict the accuracy of the model, as illustrated in Figure 7.

(7) $$\begin{array}{l} \sigma =\frac{1}{\alpha (\varepsilon )}\ln\{Z/A{\left(\varepsilon \right)}^{\frac{1}{n(\varepsilon )}}+[(Z\;/A{\left(\varepsilon \right)}^{\frac{2}{n(\varepsilon )}}+1{]}^{\frac{1}{2}}\}\\ \;\;\;\;\;Z=\dot{\varepsilon }\cdot \exp(\frac{Q(\varepsilon )}{\mathrm{RT}}) \end{array}$$

**Figure 8:**
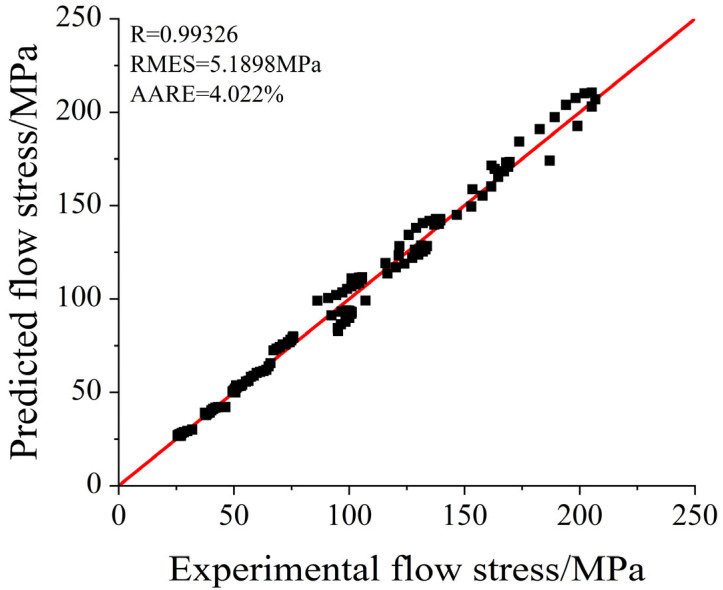
Comparison of predicted and experimental stress–strain curves for the strain-compensated Arrhenius model.

To further evaluate the accuracy and applicability of the constitutive model in predicting the thermal deformation behavior of C83600, metrics such as the correlation coefficient R, root mean square error RMES, and average relative error AARE were introduced. These metrics gauge the correlation between experimental values and model predictions, as expressed in Equations (8)–(10).

(8) $$R=\frac{\sum_{i=1}^{i=N}\left(\sigma_{\exp}^{i}-{\bar{\sigma }}_{\exp})(\sigma_{P}^{i}-{\bar{\sigma }}_{P}\right)}{\sqrt{\sum_{i=1}^{i=N}{\left(\sigma_{\exp}^{i}-{\bar{\sigma }}_{\exp}\right)}^{2}\sum_{i=1}^{i=N}{\left(\sigma_{P}^{i}-{\bar{\sigma }}_{P}\right)}^{2}}}$$

(9) $$\mathrm{RMES}=\sqrt{\frac{1}{N}{\left(\sigma_{\exp}^{i}-\sigma_{P}^{i}\right)}^{2}}$$

(10) $$\mathrm{AARE}=\frac{1}{N}\sum_{i=1}^{N}\left|\frac{\sigma_{\exp}^{i}-\sigma_{P}^{i}}{\sigma_{\exp}^{i}}\right|\times 100\%$$

where s_exp_ is the experimental flow stress, s_P_ is the predicted flow stress from the constitutive model, N is the number of data points, and $\bar{\sigma }$_exp_ and $\bar{\sigma }$_p_ are the average values of s_exp_ and s_P_, respectively. A higher correlation coefficient does not necessarily indicate that the constitutive model is suitable for all deformation conditions. It primarily reflects the trend of flow stress changes within the plastic region [19,20,21]. The calculated values of R, RMES, and AARE were 0.99326, 5.1898, and 4.022%, respectively, as depicted in Figure 8. This demonstrates that the Arrhenius constitutive model provides a high correlation and low error, confirming its suitability for describing the thermal deformation behavior of C83600 under specified conditions.

**Figure 9:**
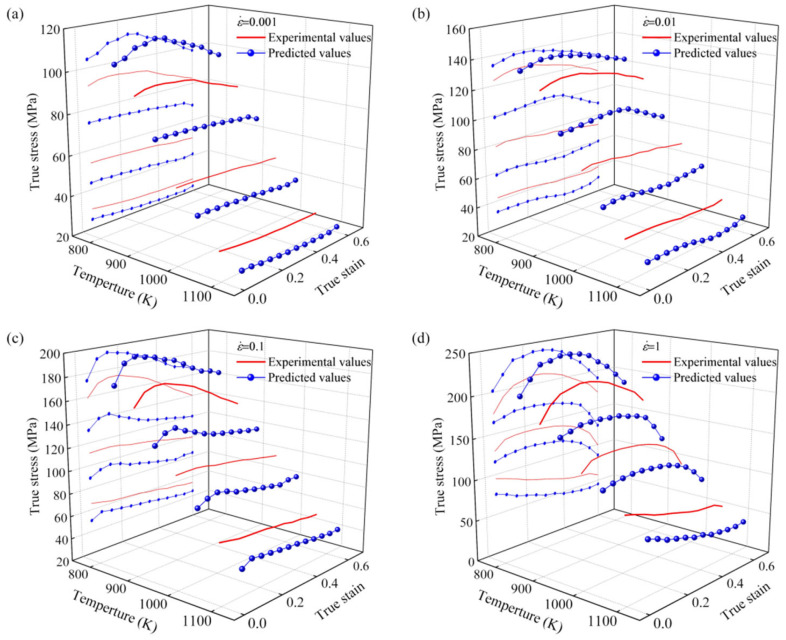
Expanded stress–strain data: (**a**) $\dot{\varepsilon }$ = 0.001 s^−1^; (**b**) $\dot{\varepsilon }$ = 0.01 s^−1^; (**c**) $\dot{\varepsilon }$ = 0.1 s^−1^; (**d**) $\dot{\varepsilon }$ = 1 s^−1^.

The above research indicates that the strain-compensated Arrhenius model exhibits high prediction accuracy; therefore, it is necessary to expand the dataset for the high-temperature flow stress of C83600. The deformation conditions were set as follows: temperatures of 823 K, 923 K, 1023 K, and 1123 K; strain rates of 0.001 s^−1^, 0.01 s^−1^, 0.1 s^−1^, and 1 s^−1^; and strain ranging from 0.05 to 0.65. The expanded stress–strain data, illustrated in Figure 9, show a consistent variation in the flow stress curve, indicating that the predicted data are reliable. This expanded dataset provides crucial support for the development of subsequent hot working diagrams and the numerical simulation analysis of the forging and forming of C83600, which is essential for devising and optimizing deformation technologies and process parameters.

### 3.3. Construction of Hot Working Diagram

The dynamic material model, proposed by Gegel and Prasad et al., is grounded in the principles of large plastic deformation, continuum mechanics, physical system simulation, and irreversible thermodynamics. It accounts for thermodynamically closed systems during forging, highlighting the significance of energy exchange and dissipation among the equipment, die, and workpiece [22,23]. The power dissipation diagram reveals the mechanisms of microstructure evolution during the hot deformation of alloy materials, such as dynamic recovery and dynamic recrystallization. The ratio of the dissipation covariance to its maximum value is represented by the power dissipation factor *η*, which is expressed as Equation (11). *η,* as calculated from the equation, facilitates the creation of a contour map of the alloy material under various deformation conditions, forming its power dissipation diagram.

(11) $$\eta =\frac{J}{J_{\max}}=\frac{2m}{m+1}$$

In general, a higher power dissipation factor *η* increases the likelihood of dynamic recrystallization and superplasticity, enhancing the microevolution mechanism of the microstructure. This suggests better processing properties under such deformation conditions. However, studies have shown that even in regions with a high power dissipation factor, instabilities such as cracks and local flow can still occur [24]. The Prasad instability criterion, the most widely utilized at present, is employed to assess plastic instability in each region of C83600. This criterion is detailed in Equation (12), and the *x* derived from this equation is used to generate a contour map of the alloy material under different deformation conditions, thereby forming its instability map.

(12) $$\xi (\dot{\varepsilon })=\frac{\partial \ln(\frac{m}{m+1})}{\partial \ln\dot{\varepsilon }}+m<0$$

**Figure 10:**
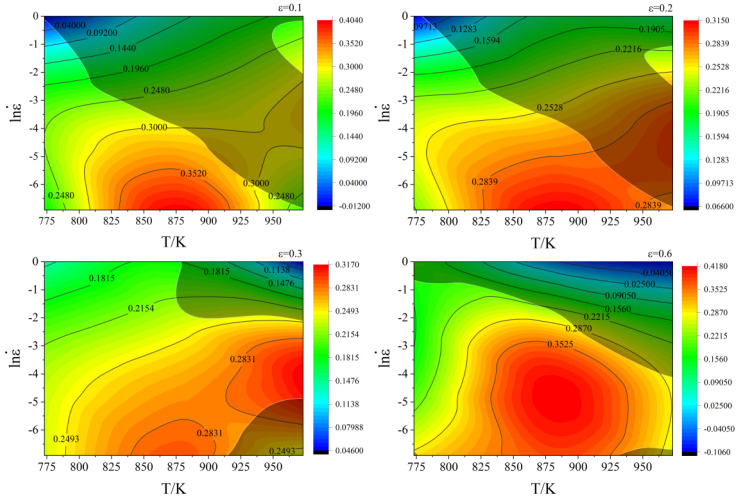
Hot working diagram of C83600 under different strain conditions.

Finally, the power dissipation diagram and instability diagram are superimposed to construct the hot working diagram, with temperature plotted on the X-axis and ln(ε) on the Y-axis under strains of 0.1, 0.2, 0.3, and 0.6, as illustrated in Figure 10. This diagram highlights the dissipative instability characteristics of cast C83600 under various strains, where the solid black line represents the isoline of *η*, and different colored regions indicate different values of *η.* The gray shadowed area denotes the plastic instability zone, and areas outside this zone are considered safe. It has been observed that higher deformation temperatures and lower strain rates increase *η* [25]. Taking into account the deformation temperature, strain rate, *η*, and instability coefficient, optimal thermal processing parameters for C83600 are established: temperatures between 850 and 900 K and strain rates between 0.0067 and 0.0483 s^−1^.

### 3.4. Valve Body Forging Simulation Verification

**Figure 11:**
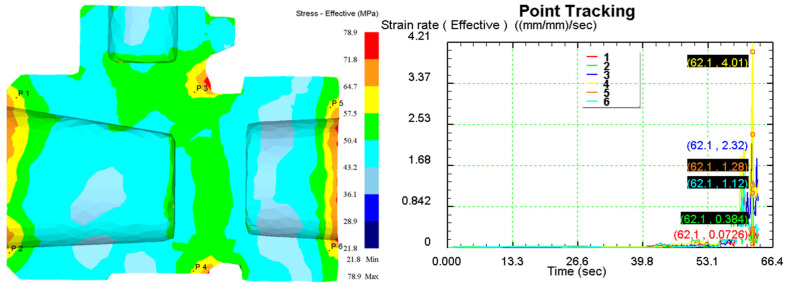
Stress and strain rate cloud diagram by the end of the forging forming.

Figure 11 displays the stress and strain rate cloud diagram of the C83600 valve body at the end of forging under the optimal process parameters derived from the hot working diagram. Six points were selected for point tracing at both ends of the valve body and the junction of the branch pipe to generate their time-strain rate curves. In the early stages of forming, the strain rates at each point are low, increasing sharply at 62.1 s, indicating the final shaping stage where the forming force peaks. Based on the time-strain rate curves, the average strain rate during the forming process at each point is calculated, as shown in Table 4. The maximum average strain rate is 0.0386 at point 4, located at the junction of the branch pipe, suggesting that alloy flow is most challenging there. The minimum average strain rate is 0.00889 at point 1, located at the left end. These values are consistent with the optimal strain rates (0.048–0.0067 s^−1^) from the hot working diagram, confirming the validity of the optimal process parameters.

### 3.5. Tin Bronze Valve Body Trial Production

**Figure 12:**
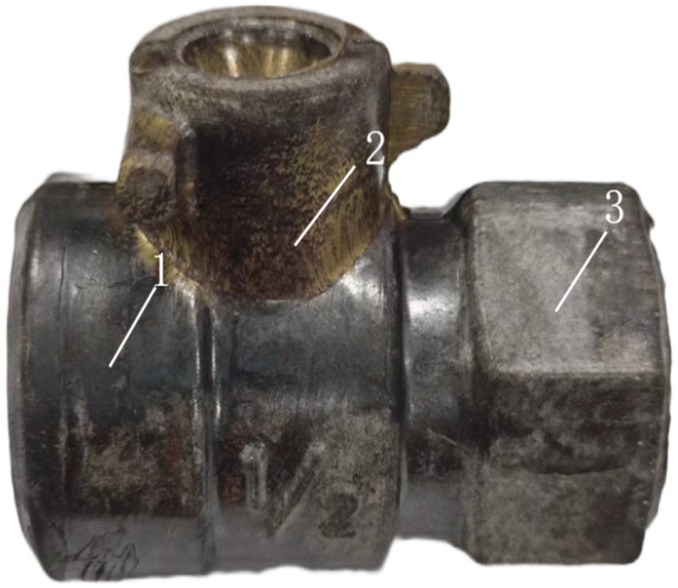
Valve body forging.

**Figure 13:**
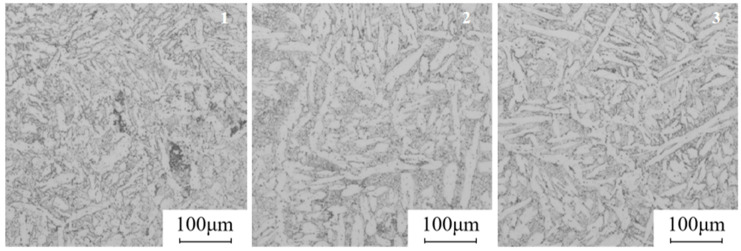
Microstructure of different regions of the forging.

The valve body forging produced using simulation parameters is shown in Figure 12. The forging is completely filled with no signs of underfilling and displays only a minor forging flash bump that does not impact machining. Overall, the surface of the forging is smooth, with no material adhering to the mold and no defects such as folding observed, indicating that the forging meets the design requirements. Samples 1, 2, and 3 were taken from the three areas of the forging, which are the three branch locations of the valve body, as shown in Figure 12, were analyzed for microstructure, depicted in Figure 13. The grain size in each region is small and relatively uniform, demonstrating that the mechanical properties of the valve body can be effectively enhanced. This also verifies that a fine and uniform microstructure can be achieved using the recommended forging temperature and strain rate from the hot working diagram.

## 4. Conclusions

The traditional casting of C83600 tin bronze valve bodies fails to meet performance requirements and service demands in high-pressure and corrosive conditions over prolonged periods. Forging significantly enhances mechanical properties and air tightness. This research examines its rheological data, Arrhenius constitutive model, and thermal processing diagram under various deformation conditions, leading to the following conclusions:

(1) Flow stress curves for C83600 were obtained via thermal simulation experiments conducted at various deformation temperatures and strain rates. Notably, when the temperature surpasses 973 K, the flow stress curve reaches its peak, indicating significant softening due to the dynamic recrystallization effect, which substantially outweighs work hardening.
(2) Using the hot working diagram, optimal forging process parameters were identified: temperatures ranging from 850 to 900 K and strain rates varying from 0.0067 to 0.0483 s^−1^. These conditions promote high thermal stability during working, enhance dynamic recovery and recrystallization processes, and facilitate the formation of a fine and uniform microstructure.
(3) The valve body forging produced using simulation parameters is completely filled with no signs of underfilling and displays only a minor forging flash bump. The feasibility of the forging process was further substantiated by trial production, which also verified that the microstructural outcomes align with the forging temperatures and strain rates recommended by the hot working diagram.

## Figures and Tables

**Table 1:** Chemical composition (%) of C83600 tin bronze alloy.

Chemical Composition	Cu	Sn	Zn	Pb	Fe	Al	Te	Si	Ni	P	S
Content (%)	Bal	5.0	5.0	5.0	0.3	0.01	0.25	0.01	2.5	0.05	0.1

**Table 2:** Boundary conditions for the forging of the tin bronze valve.

Parameters	Value
Extruding velocity (mm/s)	0.2
Initial forging temperature (K)	923
Mold temperature (K)	573
Heat conductivity coefficient (N/s/mm/°C)	4
Friction coefficient	0.2
Blank Material	C83600
Mold material	AISI-H-13

**Table 3:** Fitting coefficients of quintic polynomial for each material constant: α, n, Q, lnA.

	α		***n***		**Q**		**lnA**
$B_{0}$	0.01116	$C_{0}$	6.35582	$D_{0}$	249.69938	$E_{0}$	26.96271
$B_{1}$	0.00697	$C_{1}$	−13.19679	$D_{1}$	751.24089	$E_{1}$	80.17521
$B_{2}$	−0.06228	$C_{2}$	31.25312	$D_{2}$	−6339.93351	$E_{2}$	−686.977201
$B_{3}$	0.36319	$C_{3}$	63.75671	$D_{3}$	28746.06675	$E_{3}$	3155.2434
$B_{4}$	−0.76296	$C_{4}$	−314.68161	$D_{4}$	−57157.04415	$E_{4}$	−6328.19644
$B_{5}$	0.54751	$C_{5}$	307.03771	$D_{5}$	40853.68326	$E_{5}$	4553.59336

**Table 4:** Average strain rate of each point forging forming.

Number	Point 1	Point 2	Point 3	Point 4	Point 5	Point 6
Value	0.00889	0.01129	0.0234	0.0386	0.0152	0.0160

## Data Availability

The original contributions presented in this study are included in the article. Further inquiries can be directed to the corresponding authors.
